# A Critical Analysis of the Scientific and Commercial Rationales for the *De Novo* Synthesis of Horsepox Virus

**DOI:** 10.1128/mSphere.00040-18

**Published:** 2018-03-07

**Authors:** Gregory D. Koblentz

**Affiliations:** a Schar School of Policy and Government, George Mason University, Arlington, Virginia, USA

**Keywords:** DNA synthesis, biosecurity, dual use research, horsepox, smallpox, synthetic biology, vaccines

## Abstract

This article evaluates the scientific and commercial rationales for the synthesis of horsepox virus. I find that the claimed benefits of using horsepox virus as a smallpox vaccine rest on a weak scientific foundation and an even weaker business case that this project will lead to a licensed medical countermeasure. The combination of questionable benefits and known risks of this dual use research raises serious questions about the wisdom of undertaking research that could be used to recreate variola virus. This analysis also raises important questions about the propriety of a private company sponsoring such dual use research without appropriate oversight and highlights an important gap in United States dual use research regulations.

## Abstract (precis)

This article evaluates the scientific and commercial rationales for the synthesis of horsepox virus. I find that the claimed benefits of using horsepox virus as a smallpox vaccine rest on a weak scientific foundation and an even weaker business case that this project will lead to a licensed medical countermeasure.

## COMMENTARY

On 2 March 2017, the United States biotech firm Tonix Pharmaceuticals became the latest company to join the biodefense industry when it announced that it was developing a new smallpox vaccine called TNX-801 (1). Unlike other smallpox vaccines that are based on vaccinia virus, TNX-801 is based on a strain of horsepox virus that has been considered extinct for several decades. With modest funding provided by Tonix, scientists at the University of Alberta used the genetic sequence of a horsepox virus that had been isolated from a horse in Mongolia in 1976 to recreate the virus in their lab (2). This research represents the first *de novo* synthesis of a member of the *Orthopoxvirus* genus, a closely related group of viruses that also includes vaccinia virus and variola virus, the causative agent of smallpox. The applicability of these methods to the synthesis of variola virus, which has been eradicated from nature and is known to exist in only two WHO-designated laboratories, has raised serious questions about the risks posed by this research (3, 4). As is the case with all examples of dual use research that have peaceful applications but could also be misused to cause harm, it is necessary to critically assess both the risks and the benefits of such research. So far, Tonix’s claims about the potential benefits of synthesizing horsepox virus for the purpose of creating a smallpox vaccine have gone unchallenged (5).

This article evaluates Tonix’s scientific and commercial rationales for synthesizing horsepox virus. While this research may have utility for investigating viral genomics or developing new oncolytic agents, Tonix has justified this work on the grounds that it will lead to the production of a safer smallpox vaccine. I find that the claimed benefits of using horsepox virus as a smallpox vaccine rest on a weak scientific foundation and an even weaker business case that the company can convert this project into a licensed medical countermeasure. The combination of questionable benefits and known risks of this research raises serious questions about the propriety of a private company sponsoring such dual use research without appropriate oversight. While a retrospective analysis of the rationale and expected benefits of this research is not ideal—this type of analysis should have been undertaken by an independent, multidisciplinary group of experts before the research was conducted—it serves to highlight important gaps in U.S. oversight of dual use research that need to be closed to prevent future such biosecurity failures.

## EVALUATING THE SCIENTIFIC AND COMMERCIAL RATIONALES FOR SYNTHESIZING HORSEPOX VIRUS

According to Seth Lederman, CEO of Tonix, “Presently, the safety concern[s] of existing smallpox-preventing vaccines outweigh the potential benefit to provide immunization of first responders or the general public. By developing TNX-801 as a horsepox vaccine to prevent smallpox infection, we hope to have a safer vaccine to protect against smallpox than is currently available.” (1) Tonix’s belief that horsepox virus would provide a safer alternative to the vaccinia-based smallpox vaccines currently in use is based on the premise that the original smallpox vaccine pioneered by Edward Jenner was actually based on horsepox virus, not cowpox virus as commonly believed. According to this theory, as the virus used for vaccination was selected for properties that favored large-scale production over the years, it evolved into vaccinia virus. As horsepox virus evolved into vaccinia virus, it also acquired phenotypic changes that caused serious adverse side effects, such as the cardiotoxicity that was seen during the 2002–2003 smallpox immunization campaign in the United States. Thus, according to Tonix, an ancestral strain of vaccinia virus—namely, the horsepox virus—would not have these undesirable properties (1, 6).

The putative benefit to synthesizing horsepox virus for use as a smallpox vaccine therefore rests on four assumptions made by Tonix: that the modern-day smallpox vaccine based on vaccinia virus is directly descended from horsepox virus, that ancestral horsepox virus is a safer candidate for a human vaccine than derived vaccinia virus, that current smallpox vaccines are not safe enough, and that there is a significant demand for a new smallpox vaccine. All four of these scientific and commercial claims need to be true to fully realize the expected benefit of synthesizing horsepox virus. I argue that there are serious doubts that all of these assumptions are valid, raising important questions about the wisdom of synthesizing this virus given the risks posed by pioneering a technique that could be used to recreate variola virus.

The strain of horsepox virus that was synthesized, which was isolated from a horse in Mongolia in 1976, is almost certainly not the directly ancestral strain of the virus that Edward Jenner used in the 1790s. In addition, the genetic makeup of horsepox virus does not provide a clear *a priori* indication that this virus is inherently better suited for being used as a human vaccine than vaccinia virus. Furthermore, there is no viable business model for developing a new smallpox vaccine based on a novel viral platform. While first- and second-generation smallpox vaccines were associated with a significant rate of adverse side effects, the third-generation smallpox vaccines that are currently available have very good safety profiles. Tonix’s business model for developing a horsepox-based smallpox vaccine depends entirely on U.S. government support for this new medical countermeasure to make it through the so-called “valley of death” in the drug development process. The U.S. government, however, has expressly stated that it is not interested in funding the development of a new smallpox vaccine. Given the weak scientific rationale and poor business case for synthesizing horsepox virus for the purposes of developing a new smallpox vaccine, the purported benefits of this research will likely remain illusory.

## WEAK SCIENTIFIC BASIS FOR HORSEPOX VIRUS STRAIN MNR-76 AS ANCESTRAL STRAIN FOR VACCINIA

While horsepox virus, or a vaccinia-like virus that infected horses in the 18th and 19th centuries, may have been a component of early smallpox vaccines, the MNR-76 strain that was recently synthesized is most likely not directly ancestral to vaccinia virus. Instead, both MNR-76 and vaccinia virus are more likely descended from a common, but currently uncharacterized ancestor. Determining the origin of vaccinia virus, and its relationship to horsepox virus, is complicated by the primitive state of scientific knowledge during Jenner’s time, the varied and largely undocumented vaccine cultivation practices of early vaccinologists, and the existence of only a single sequenced strain of horsepox virus for phylogenetic comparison.

There is good historical evidence that early vaccinators used virus obtained from horses, cows, and humans, and sometimes a mixture of these, to induce immunity against smallpox (7, 8). Scientists during Jenner’s era, however, could not identify the causative agent for pustular diseases in cows and horses. As a result, pustular diseases in cows at that time were referred to as cowpox, while similar diseases in horses were referred to as horsepox. These diseases could have been caused by what we now call vaccinia virus, horsepox virus, or cowpox virus (9). Since different poxviruses can cause similar looking diseases in the same type of animal, the type of animal used as the source of virus to produce early smallpox vaccines does not provide reliable insights into the identity of the virus.

Phylogenetic analyses of orthopoxviruses have also been used to investigate the origins of vaccinia viruses and the relationships within this family of viruses. There is strong phylogenetic evidence that horsepox virus and vaccinia virus are closely related (8, 10). There is not enough evidence, however, to demonstrate that horsepox virus is directly ancestral to vaccinia virus. The 2006 *Journal of Virology* study by lead author Edan Tulman that sequenced the MNR-76 strain does find that horsepox and vaccinia viruses are very closely related, lending support to the theory that MNR-76 and vaccinia viruses share a relatively recent common ancestor (11). At the same time, Tulman presents greater genetic distance (i.e., longer branch length relative to those of other viruses in the vaccinia virus family tree), suggesting that MNR-76 has been evolving separately from a common ancestor for some time. The original study reports that “while very closely related, HSPV [horsepox virus] is phylogenetically distinct from other characterized VACV [vaccinia]-like viruses.” (11) More recent analyses with additional vaccinia strains support this finding (8, 10).

The recent analysis of a 1902 strain of smallpox vaccine, which reportedly has the highest degree of similarity to MNR-76, has been presented as evidence that horsepox virus was used to create early smallpox vaccines (12). The reliability of this finding is unclear, however, because the article only presents pairwise comparison between the core genomes of the 1902 vaccine and MNR-76 and does not compare the two in the context of other, closely related orthopoxviruses. The article also makes clear that the 1902 vaccine strain does not contain large terminal genomic region sequences present in MNR-76, consistent with deletion of this sequence from other known vaccinia viruses.

There are unique aspects of MNR-76 that cast further doubt on the hypothesis that this strain of horsepox virus is the directly ancestral strain for vaccinia virus. Most importantly, the horsepox genome contains multiple fragmented genes that are intact in all, or nearly all, other vaccinia-like viruses (11). This finding indicates that the MNR-76 strain of horsepox virus has certain features that are evolutionarily more recent (i.e., derived, or nonancestral) than those in the vaccinia viruses previously sequenced. According to Tulman, “Despite speculation as to what role horsepox played in the development of smallpox vaccines, it is clear that HSPV MNR-76 does not represent a direct ancestral genotype to all known VACVs, given the disruption of many HSPV genes intact in certain VACV isolates.” (11) Since the MNR-76 strain was obtained from a relatively recent outbreak in Asia, it is highly unlikely to have been the same strain used by Jenner and other vaccinologists in Europe 200 years earlier.

It is unclear where the horsepox virus involved in the 1976 outbreak in Mongolia originated from. One hypothesis, common for other vaccinia-like viruses isolated from domestic animal species, is that the horsepox seen during the 1976 outbreak was the result of a currently uncharacterized vaccine strain of vaccinia virus that was used and escaped while mass immunization against smallpox was common and perhaps which circulated among animals in the wild before causing the 1976 outbreak (11). Similar cases of vaccinia-like diseases in animals caused by human-transmitted vaccinia infections have been tied to the emergence of buffalopox in India. A recent article provides additional perspective to the concept of vaccine diversity and vaccine escape, as the historical IOC vaccine strain used in Brazil appears to be part of a distinct phylogenetic cluster, including wildly circulating, and apparently derived, vaccinia viruses, but also including the long-branching MNR-76 strain (8).

Another possibility is that the virus that causes the disease we call horsepox is not endemic to horses, but is a vaccinia-like virus that has another animal as its reservoir. In that scenario, the 1976 outbreak was the result of a naturally circulating orthopoxvirus that jumped from the reservoir species to horses. For example, wild rodents are now known to be the primary reservoir host species for cowpox, and they have served as the source for outbreaks in other species of wild, domesticated, and zoo animals in Europe (13).

A recent review of the history of smallpox vaccines summarized the difficulty of conclusively demonstrating the origins of vaccinia virus and its relationship to horsepox virus, “Notably, all phylogenetic studies are based on a single existing sample of horsepox virus, and it is unknown whether this 1976 virus represents a true autochthonous strain or that it might be a vaccine escapee. In any case, its genome has certainly evolved and diverged from ancient 18th and 19th century horsepox viruses” (9). Thus, it is highly unlikely that synthesizing a strain of horsepox virus isolated from a horse in Mongolia in 1976 would provide a genetic copy of the original smallpox vaccine used by Jenner in 1796.

## WEAK SCIENTIFIC BASIS THAT HORSEPOX VIRUS WOULD BE SAFER ALTERNATIVE FOR HUMAN VACCINE USE

Another important factor to consider is whether what is known about the horsepox virus genome supports the claim that this virus would provide a safer alternative to vaccinia virus for use as a vaccine. Although there are important gaps in our understanding of the roles and functions of different *Orthopoxvirus* genes and the relationship between genotype and phenotype, there are some indicators that the genetic structure of horsepox virus does not provide confidence that the virus would cause fewer side effects than vaccinia virus. Most importantly, horsepox virus has more orthopox genes in it than does vaccinia virus, and some of these genes are associated with virulence and host range in other orthopoxviruses. MNR-76 contains seven full-length genes that are fragmented or missing in other vaccinia-like viruses, including intact homologues of the cowpox strain GRI-90 D2L/I4R CrmB and D13L CD30-like tumor necrosis factor receptors that are used to manipulate the host immune system, D3L/I3R and C1L ankyrin repeat and B19R Kelch-like proteins that potentially allow the virus to better survive in specific cell types and hosts, and the B22R protein (11). Notably, several of the genes that are intact in MNR-76, but absent from known vaccinia viruses, have homologues that positively affect viral virulence and host range in other poxviruses, essentially contributing to the ability of the virus to cause disease. For example, in other orthopoxviruses, homologues of B22R inactivate host immune cells (14). Thus, based on our incomplete knowledge of *Orthopoxvirus* genomics, virulence, and host range, there does not appear to be a strong scientific rationale for believing that horsepox virus would be better suited than vaccinia virus for use as a human vaccine.

## NO DEMONSTRATED NEED FOR A SAFER SMALLPOX VACCINE

The third assumption by Tonix—that a safer smallpox is needed—is not supported by the evidence. While the first-generation Dryvax vaccine, and its successor, ACAM2000, did exhibit higher-than-expected rates of serious cardiac events during the 2002–2003 smallpox immunization campaign, currently available third-generation vaccines do not cause this adverse side effect. These third-generation vaccines are already being stockpiled by the United States, Japan, and Canada.

During the 2002–2003 smallpox immunization program in the United States, a serious new cardiac complication—myopericarditis—emerged (15). Of the 39,213 civilians immunized with the Dryvax vaccine, there were 16 suspected cases and 5 probable cases of myopericarditis, including 3 deaths (16). Among the almost 500,000 military personnel immunized with Dryvax, 58 were identified with confirmed or probable acute myopericarditis (17). This adverse effect was likely not new, but was only recognized in 2002 due to better surveillance of vaccine adverse events and advances in diagnostics needed to confirm cases of myopericarditis, which typically has mild and transient symptoms (18). Nonetheless, the emergence of this unexpected and potentially deadly side effect effectively derailed the civilian immunization campaign. The second-generation ACAM2000 live vaccine, based on the same strain as Dryvax but produced in a cell culture, has a similar safety profile to Dryvax, including incidence of myopericarditis (19). The ACAM2000 vaccine was licensed by the Food and Drug Administration (FDA) in 2007 and has replaced Dryvax in the U.S. stockpile (20).

The safety issues associated with first- and second-generation smallpox vaccines led to the development of more highly attenuated, third-generation vaccines, such as modified vaccinia Ankara (MVA) and LC16m8, that do not exhibit the same safety problems as previous vaccines. Bavarian Nordic has developed a live, highly attenuated nonreplicating smallpox vaccine based on MVA, called Imvamune or Imvanex, which has been shown to be safe and well tolerated. The vaccine does not present the risk of myopericarditis that was observed with Dryvax and ACAM2000 and can even be given to populations who could not receive second-generation smallpox vaccines, such as those infected with HIV, with compromised immune systems, or with certain skin conditions (21, 22). The vaccine has been approved for use by the EU under the name Imvanex and by Canada under the name Imvamune (23). The United States has stockpiled 24 million doses of the vaccine under the name Imvamune for use during a public health emergency (24). In September 2017, the United States signed a contract with Bavarian Nordic for up to 132 million additional doses of a freeze-dried version of the vaccine (25).

The LC16m8 attenuated, replicating smallpox vaccine developed in Japan has proven to be safe and effective, without the cardiotoxicity associated with Dryvax and ACAM2000. LC16m8 has been described as “one of the safest live, attenuated, replication-competent vaccines” (7). While the vaccine is not currently approved for use in patients with HIV, compromised immune systems, or generalized skin diseases, Japanese scientists are conducting research with animal models to demonstrate the vaccine’s safety in such populations (26). LC16m8 is the sole smallpox vaccine licensed and stockpiled in Japan and has investigational new drug (IND) status in the United States (27). The WHO’s Strategic Advisory Group of Experts on Immunization has recommended LC16m8 for inclusion in the WHO’s smallpox vaccine stockpile (28).

## NO DEMAND FOR A NEW SMALLPOX VACCINE

The United States, the only market large enough to justify development of a new smallpox vaccine, has clearly indicated that it is not interested in investing in new types of smallpox vaccines. Without funding from U.S. Government sources, there is little prospect that Tonix will be able to develop TNX-801 into a licensable medical countermeasure.

In 2017, the United States spent $1.6 billion on biodefense programs designed to counter biological weapons as part of a broader health security budget of $13 billion (29). As a result, the United States accounts for more than half of the global biodefense market (30). Although several other countries have stockpiles of smallpox vaccine, the majority of these stockpiles consist of first-generation vaccines that had been used during the smallpox eradication campaign (31). Since 2001, only a handful of countries are publicly known to have assessed the threat of smallpox as being severe enough to warrant investing in more advanced smallpox vaccines. The United States presents the largest market for smallpox vaccine, with a standing requirement to stockpile enough second-generation vaccine for 300 million people and enough third-generation vaccine for 66 million people (32). In 2002 to 2003, Bavarian Nordic sold 100 million doses of the second-generation Elstree-BN vaccine, based on the Lister-Elstree vaccine strain used during the global eradication campaign, to Germany and 20 million doses to the United Kingdom (33–35). Japan has a stockpile of 30 million doses of the LC16m8 vaccine (36). Singapore has licensed the use of ACAM2000 and has stockpiled enough vaccine for its entire population of under 6 million people (37). In 2004, Australia approved the purchase of 200,000 doses of ACAM2000 (34). Canada has purchased 540,000 doses of Imvamune (38, 39). Other countries that stockpile first-generation vaccines, such as France, have identified a need for a safer vaccine but have been unwilling to commit the resources necessary to procure this new medical countermeasure (40). The small size of most of these national stockpiles means that the global smallpox vaccine market is highly fragmented.

Due to higher financial and liability risks associated with biodefense vaccines and the unfavorable rate of return on this type of product compared to other drugs with larger markets, government support has historically played a crucial role in developing these medical countermeasures (41). A new smallpox vaccine would be no different. D. A. Henderson has estimated that the cost of developing a new smallpox vaccine and production facility would be between $750 million and $1.75 billion (42). A new smallpox vaccine will require government funding to support the research and development necessary to achieve licensure and large-scale procurement contracts to justify the construction of a new production facility. Since 2012, however, the Public Health Emergency Medical Countermeasure Enterprise (PHEMCE), the interagency body in the United States charged with coordinating the development and stockpiling of new medical countermeasures, has reported that the existing smallpox vaccines are mature and that it “will seek to focus any future research investments in these areas on improvements to the current capabilities rather than development of new capabilities” (43, 44). The focus of the Biomedical Advanced Research and Development Authority (BARDA), the organization within the Department of Health and Human Services responsible for developing new medical countermeasures, has been on the development of a freeze-dried formulation of Imvamune with a longer shelf-life to reduce life cycle management costs. In September 2017, BARDA awarded a 5-year contract, worth potentially $529 million, to Bavarian Nordic to supply up to 132 million doses of this improved vaccine to the stockpile and obtain FDA licensure for use with the general population (45).

In promotional material for potential investors, Tonix cites two ways that the horsepox vaccine could generate profits for the company: by obtaining a priority review voucher (PRV) that they could sell to another company or by selling the vaccine candidate itself to another company (6). The business case for both strategies, however, is weak since they both rely on financial support from the U.S. Government which is unlikely to be forthcoming.

As outlined in a company document, Tonix plans on taking advantage of a new law designed to encourage the development of new biodefense medical countermeasures to obtain a PRV once the FDA licenses TNX-801. Under section 3086 of the 21st Century Cures Act passed in December 2016, the FDA will award a PRV to a company upon approval of a new drug application for a medical countermeasure against a pathogen designated as a material threat. The PRV entitles the owner to receive a review of their new drug application within 6 months compared to the usual 10 months. The PRV may be used by the sponsor who receives it or sold to another sponsor, who may then use it to obtain priority review for a product application that would otherwise not receive priority review (46). A horsepox virus-based smallpox vaccine would be eligible to receive a PRV since smallpox is listed as a material threat by HHS and it contains an active ingredient that has not been previously approved by the FDA.

Tonix, however, will face several challenges in capitalizing on the incentives contained in the 21st Century Cures Act. First, section 3086, which authorizes this PRV, expires on 1 October 2023. Since the average time it takes for vaccines to move from basic research to licensed product is 10 to 15 years, it is highly unlikely that a horsepox virus-based smallpox vaccine will be eligible for a PRV before this section of the law expires (47). Second, Tonix overestimates the value of future PRVs. According to Tonix, PRVs in the past have been sold for as much as $125 million (6). As more PRVs are issued, however, the value of these vouchers is likely to decline. Third, even if section 3086 is extended and the value of PRVs can be sustained for the 10 to 15 years it will take Tonix to complete the FDA licensure process, the company faces a major hurdle in preparing a new drug application for a new smallpox vaccine based on horsepox virus. In order to submit a new drug application, companies must collect data on the efficacy of the drug in animals and the safety of the drug in humans and provide information to the FDA on the production processes used to manufacture the drug. These activities are largely located in the so-called “valley of death” in the drug development process, where costs and risks increase significantly. The majority of the costs of vaccine development are incurred at this time due to the need to run large clinical trials and begin scaling up production processes. At the same time, only 20% of drugs that enter phase I clinical trials will be licensed (41). This combination of high cost and high risk requires all but the largest biotech and pharmaceutical companies to seek outside funding and expertise to safely traverse this stage of the drug development process. BARDA was created in 2006 to help biodefense companies, which tend to be smaller biotech companies like Tonix, cross the valley of death by providing funding for advanced development that venture capital firms and pharmaceutical companies are unwilling to provide. As previously noted, since BARDA has decided that the United States smallpox vaccine stockpile is already mature, it is unlikely the agency would be willing to provide significant funding to Tonix to complete the development activities necessary for a new drug application. Without a substantial level of government funding, Tonix is likely to find it extremely difficult to ride its horsepox-based vaccine through the valley of death.

According to a presentation to potential investors, Tonix’s second strategy for developing TNX-801 into a licensed smallpox vaccine is to sell the patent pending on its synthetic horsepox virus to a company capable of bridging the valley of death on its own (6). This business model is also problematic. Tonix has cited the precedent of the biotech firm Acambis selling ACAM2000 to the pharmaceutical giant Sanofi Pasteur in 2008 for $513 million. This figure, however, dramatically overstates the value of ACAM2000. First, Sanofi paid $513 million for the entire company, which in addition to ACAM2000, had a number of other vaccine candidates under development (48). Second, ACAM2000 was a proven product with a guaranteed, long-term cash flow. By 2008, ACAM2000 had been licensed by the FDA and Sanofi was able to take over a 10-year, $425 million contract that Acambis had just signed to provide the vaccine to the CDC for the Strategic National Stockpile (49). Tonix is in a much less favorable position than Acambis was in 2008 and, as noted above, will probably not have access to funding from the U.S. Government that Acambis used to develop ACAM2000. Third, Sanofi’s recent sale of ACAM2000 to Emergent BioSolutions provides a better, although imperfect, indicator of the market value of smallpox vaccines. In July 2017, Emergent paid $125 million for the rights to ACAM2000, two production facilities, and a 10-year contract with the CDC worth $160 million (50). It is highly unlikely that an established biodefense or pharmaceutical company would pay anywhere near this amount for an experimental smallpox vaccine based on a completely new viral platform that has not been licensed and for which there are no existing large-scale production facilities or government procurement contracts. Fourth, Sanofi’s sale of ACAM2000 reflects a deeper trend among pharmaceutical companies to eschew work in the biodefense field, reducing the number of potential purchasers of TNX-801. For example, GSK, one of the few members of “Big Pharma” with a biodefense drug, recently sold raxibacumab, a monoclonal antitoxin treatment for use against anthrax, to Emergent BioSolutions (51).

## CONCLUSION

At the heart of the dual use research dilemma is the need to assess and balance the benefits and risks presented by an experiment or line of research. This is a difficult task given the largely theoretical risks posed by unknown adversaries in the future and the enticing yet uncertain benefits that the research may eventually yield. Indeed, measuring risks and benefits and weighing them can be a wicked problem that defies simple or straightforward conclusions (52). The difficulty of the task, however, does not excuse researchers, funders, or journal editors from trying to do so. While the benefits of biotechnology and life sciences research are beyond question, we should not take for granted the benefits of specific experiments or avenues of dual use research. Tonix’s claims about the benefits of synthesizing horsepox virus for the purpose of creating a smallpox vaccine have gone unchallenged, including by the Dual-Use Research Committee selected by *PLoS One* to review the manuscript before publication (53).

This article argues that the scientific and commercial rationales that Tonix has used to justify this research are weaker than they appear. This finding raises serious questions about the propriety of a private company sponsoring research with questionable benefits and known risks without any form of oversight regarding the dual use applications or biosecurity implications. The *de novo* synthesis of horsepox virus did not fall under United States or Canadian dual use research oversight at the time it was conducted. Tonix, therefore, had no legal obligation to conduct a review of this research for dual use potential or seek independent analysis of the risks and benefits of this work. Given the high degree of homology between orthopoxviruses, however, the scientists who synthesized horsepox virus knew full well that the techniques they described are directly applicable to the recreation of variola virus. David Evans, who led this research at the University of Alberta, told the World Health Organization that his synthesis of horsepox virus “was a stark demonstration that this could also be done with variola virus” (54).

The ability of this research to escape oversight illustrates a major gap in dual use research policy in the United States. The current United States dual use research oversight system applies to institutions within the United States that receive federal funding for life sciences research (55, 56). Since Tonix did not receive such funding at the time it commissioned this research, it was exempt from oversight. The exemption of life sciences research that is privately funded is a large, and growing, loophole in the oversight system. Nongovernment sources of funding, such as corporations, foundations, and individuals, are accounting for an increasingly large share of life sciences research in the United States. In 2013, federal funding for the first time accounted for less than half of national spending on scientific research (57). Given the increasing size of the bioeconomy and the growing commercialization of products generated with synthetic biology and genome editing tools, exclusion of the private sector from dual use research oversight is an increasing large loophole. The rise of crowdfunding platforms, such as Experiment and Consano, is another potential source of funding for researchers in the life sciences. For example, the “Glowing Plant” project to create bioluminescent plants received $484,000 in less than 2 months on Kickstarter (58). Since the DNA used to synthesize the horsepox virus cost only $100,000, the cost of a project to synthesize variola virus is well within the realm of a crowdfunding initiative.

The exemption of privately funded research from oversight raises two immediate concerns. First, privately funded research avoids the first line of defense against risky research: scientific peer review. Proposals submitted to federal funding agencies undergo a rigorous scientific review by federal agency staff and peer scientists to ensure that the proposed research is scientifically meritorious, will have a strong positive impact on the field, and answers an important scientific question or public health need (59). There is no guarantee that proposals for dual use research submitted to private funders will receive the same type of review. Second, while privately funded research may inadvertently enter into the domain of dual use research of concern, this exemption may also create a perverse incentive for scientists interested in conducting the riskiest or most controversial dual use research to seek out such funding to avoid the oversight attached to federal funding.

The synthesis of horsepox virus is only the latest in a series of high-profile experiments in the life sciences that has raised questions about the benefits and risks of such research. As synthetic biology increasingly powers the bioeconomy and genome editing applications in medicine, public health, agriculture, and biomanufacturing attract increasing amounts of private investment, it is necessary to ensure that all life sciences research with dual use potential, regardless of the source of funding, is conducted safely, securely, and responsibly. Extending U.S. policy on oversight of dual-use research to privately funded research to ensure that the benefits of such research outweigh its risks is just one small step toward that objective.
